# Quantitative Analysis of Biogenic Amines in Different Cheese Varieties Obtained from the Korean Domestic and Retail Markets

**DOI:** 10.3390/metabo11010031

**Published:** 2021-01-04

**Authors:** Sujatha Kandasamy, Jayeon Yoo, Jeonghee Yun, Han Byul Kang, Kuk-Hwan Seol, Jun-Sang Ham

**Affiliations:** Animal Products Research and Development Division, National Institute of Animal Science, Rural Development Administration, Wanju 55365, Korea; sujirda2019@korea.kr (S.K.); yjy1172@korea.kr (J.Y.); rsvped@korea.kr (J.Y.); khb1771@hanmail.net (H.B.K.); seolkh@korea.kr (K.-H.S.)

**Keywords:** biogenic amines, cheese, high-performance liquid chromatography with a diode-array detector (HPLC-DAD), toxicity, risk assessment

## Abstract

To evaluate the safety and risk assessment of cheese consumption in the Republic of Korea, sixty cheese samples purchased from the farmstead and retails markets (imported) were analyzed for their biogenic amine (BA) contents. The BA profiles and quantities of eight amines (tryptamine, 2-phenylethylamine, putrescine, cadaverine, histamine, tyramine, spermidine, and spermine) were determined using high-performance liquid chromatography (HPLC). Spermine was the only amine detectable in all the samples. The BAs of fresh cheeses from both farmstead and retail markets were mostly undetectable, and comparatively at lower levels (<125 mg/kg) than ripened samples. Putrescine was undetectable in all the domestic ripened cheeses. The sum of BA levels in the imported ripened cheeses of Pecorino Romano (1889.75 mg/kg) and Grana Padano (1237.80 mg/kg) exceeds >1000 mg/kg, of which histamine accounts nearly 86 and 77% of the total levels, respectively. The tolerable limits of the potential toxic amines, histamine and tyramine surpassed in four and three imported ripened samples, respectively. Furthermore, the presence of potentiators (putrescine and cadaverine) together in samples even with a lower level of toxic amines alarms the risk in consumption. Therefore, adoption of strict hygienic practices during the entire chain of cheese production, along with obligatory monitoring and regulation of BA in cheeses seems to be mandatory to ensure the safety of the consumers.

## 1. Introduction

Biogenic amines (BAs) are toxic, non-volatile, bioactive low molecular nitrogenous organic bases with an aliphatic, aromatic, or a heterocyclic structure. These amines are produced via the catalytic action of substrate-specific microbial (mainly bacteria) decarboxylases that remove the α-carboxyl group of respective free precursor amino acids (histidine, tyrosine, ornithine, lysine, tryptophan, and phenylalanine) to yield their resultant amines (histamine, tyramine, putrescine, cadaverine, tryptamine, and β-phenylethylamine) [1,2]. Microorganisms possessing the decarboxylase activity can enter into the foods spontaneously or as starter cultures added intentionally into foods. The most commonly detected BA levels in foodstuff (<100 mg/kg), do not represent a serious risk in healthy consumers as they are detoxified by the intestinal enzymes such as monoamino oxidase (MAO) and diamino oxidase (DAO) [3,4]. While consumption of foods containing higher BA levels can create several health problems such as headache, nausea, dyspnea, oral burning, hives, hot flushes, sweating, heart palpitation, hyper- or hypotension, depending upon their intensities. BAs can enter the blood circulation system and might trigger several undesirable effects under situations of inadequate detoxification that occurs via the consumption of higher BA levels or because the intestinal detoxification system collapses or weakens due to the inhibitors of detoxification enzymes [2,5].

BAs occur in a wide range of lactic acid bacteria (LAB) fermented foods, comprising dairy products that accumulate histamine, tyramine, phenylethylamine, tryptamine, putrescine, and cadaverine as the most common ones [1,6]. The excessive amounts of histamine, tyramine, and phenylethylamine can produce detrimental effects directly, while putrescine and cadaverine can increase the toxic effects of former amines [7]. The BA levels in dairy products such as milk, yoghurts, curd- cheese, and unripened cheeses normally varies between milligrams and tens of milligrams per kg, while sometimes exceeding 2000 mg/kg [8,9]. Cheeses are the most common and highly concerned dairy products relating to the incidence of BAs. Besides food poisoning under higher BA levels, several cases of histamine/tyramine intoxication have been reported in the United States, France, and Netherlands upon subsequent cheese consumption [10,11]. Occasionally, even cheeses with lower BA levels can bring forth adverse health effects in adults administering monoamine oxidase inhibitors (MAOI) and with histamine intolerance [2,12]. Several factors such as the ripening time/methods, presence of microbiota possessing decarboxylase activities, availability of free amino acid precursors, and environmental conditions (pH, temperature, salt intensity and water activity) influence the composition and the concentration of BA [6,13,14].

In cheese production process, ripening acts as an essential step in the enhancement of flavor and texture characteristics for any cheese variety. Depending upon the degree of proteolytic activity, the microbiota involved in cheese ripening accumulates free amino acids that might be precursors in the production of BAs [3,10,15]. Both starter and non-starter LAB involved in the manufacturing process, and/or contaminating microflora in the production, and ripening environments reported widely to produce BAs. The amines such as histamine, tyramine, putrescine, and cadaverine are mainly derived from their particular amino acid decarboxylase activities by the cheese microbiota [1,6,8,16]. In smear-ripened cheese, washing of cheese surface reduced the BA contents to minimum levels; however, in unwashed-rind cheeses, the mean content of tyramine, putrescine, and cadaverine surpassed 100 mg/kg [17]. Higher ripening temperatures favor the accumulation of tyramine, putrescine, and cadaverine that possess higher threat to consumer’s health. It is eminent that the concentration of amines differs in various portions of cheese with higher in edges, while lower in cores influenced by the microbial diversity at each layer [18]. 

The domestic cheese production in the Republic of Korea (ROK) is increasing over the past few years to meet the demand on consumption under both consumer market and in food processing sectors. This increasing demand is also met by several cheese varieties imported from the U.S., European Union, New Zealand, and Australia [19]. The health concerns over the food safety and healthy lifestyle forced many Koreans to consume cheeses produced from domestic farms. It is difficult for domestic manufacturers to compete against imported cheeses on price and quality [15,20]. The high popularity and demand on these products drive the assessment of their quality and health safety related to the content of BAs. No information regarding the occurrence of BAs and their levels in farmstead cheese products from the ROK is available. Several analytical procedures employing gas chromatography (GC), GC-mass spectrometry, high-performance liquid chromatography (HPLC), and HPLC-tandem mass spectrometry assessed the levels of BAs in dairy products [3,13,21]. Before HPLC analysis, a derivatization step with dansyl chloride (Dns-Cl), occurs via reactions of amino groups to boost the analytical sensitivity of the spectroscopic detectors, since most of the BAs display low UV absorption or native fluorescence under spectroscopic detection techniques [21]. The main goal toward the evaluation of BAs is to ascertain their potential role as quality indicators and to reveal the consumers about the toxicological consequences of these amines at higher concentrations [8]. In this context, the levels of eight individual (tryptamine, 2-phenylethylamine, putrescine, cadaverine, histamine, tyramine, spermidine, and spermine) and total BAs in cheese samples from the farmstead and retail markets of the ROK were analyzed using HPLC-DAD. To the best of our knowledge, this is the first study done on the risk assessment of the BA profiles and their contents in the Korean cheeses available for consumption.

## 2. Results and Discussion

**Figure 1:**
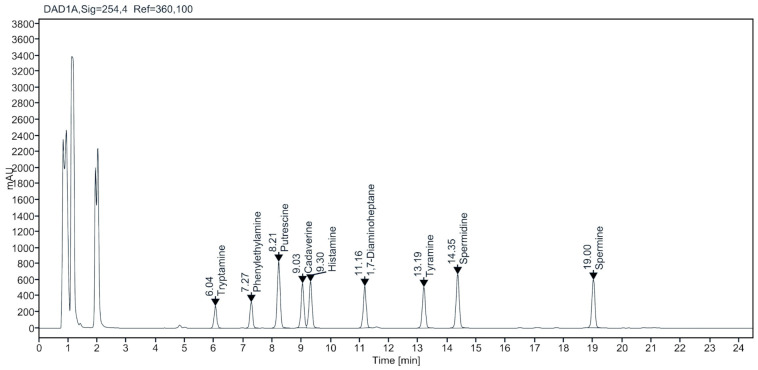
Representative **c**hromatographic separation of the biogenic amines in standard solution.

Cheese acts as an ideal substrate for production of BAs and mainly the proteolytic process during ripening accumulate free amino acids, among which some are precursors of BA. Several other factors such as the quality of raw milk, existence of microorganisms able to produce decarboxylase enzymes, duration of ripening and storage, temperature, manufacturing process, and the adopted sanitation procedures also affect the BA production in cheese [3,6,10]. In this study, the content of nine BAs in the domestic and imported cheese samples purchased from the farms and retail markets in the ROK was determined using HPLC-DAD analysis. Figure 1 illustrates the typical chromatograms from the standard mixture. The results regarding linearity, limit of detection (LOD), and limit of quantification (LOQ) are given in Table 1. The LOD and LOQ established for the cheese samples varied from 0.16 to 0.38 and 0.53 to 1.25 mg/kg respectively, and linearity ranged from 0.992 to 0.997. 

### 2.1. Diversity of the BAs in the Cheeses for Safety Assessment

Table 2 summarizes the results on the incidence of individual and total BAs in cheese samples from the farm and retail markets. In this study, we categorized the samples under four groups as domestic fresh, domestic ripened, imported fresh and imported ripened cheeses. Spermine was detected in all the 60 samples (100%). Compared with fresh samples, the detective rate of BAs was higher with ripened cheeses from both farmstead and retail markets. The domestic ripened cheeses showed a detective rate of 100% for 2-phenylethylamine, tyramine, spermidine, and spermine, while 80% for cadaverine and histamine. Whereas in imported ripened cheeses, a slight variation in the detection rate of the individual BAs was found between the hard, semi-hard, and soft-ripened types. In hard cheese type, putrescine and histamine were found in 60% samples, while 100% for cadaverine, tyramine, and spermine. Likewise, in semi-hard type cheeses the detective rate of histamine and spermine was 100%, followed by tyramine (92.9%) and cadaverine (85.7%). In soft-ripened cheeses, tyramine and spermine were detected at 100%, followed by putrescine (84.6%).

#### 2.1.1. BAs in Domestic Fresh and Ripened Cheeses

**Figure 2:**
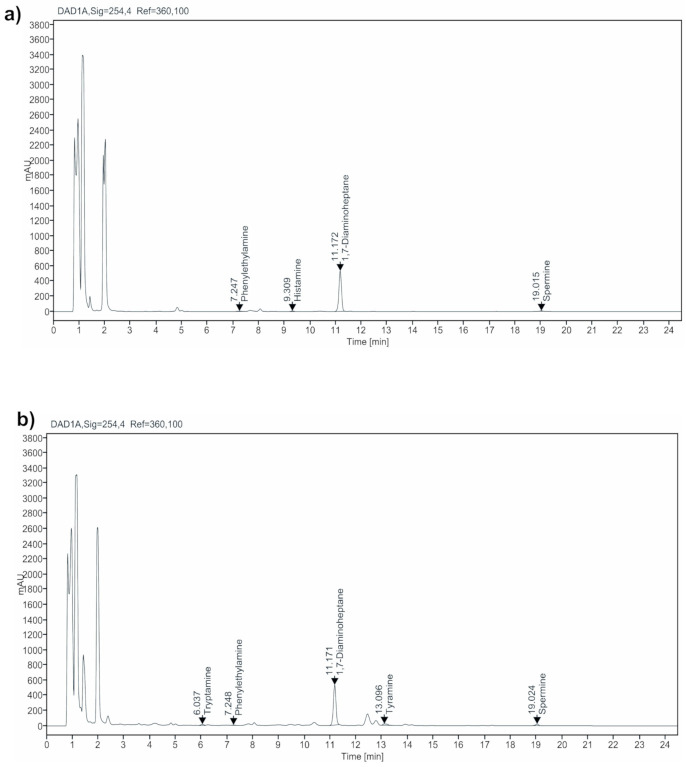
Representative chromatographic separation of biogenic amines in the fresh (**a**-string) and ripened (**b**-cheddar) cheeses from the farmsteads in the Republic of Korea.

Several investigations recommended the maximum limit to be around 50–100 mg/kg for histamine; 100–800 mg/kg for tyramine; 30 mg/kg for PHE; and 200–1000 mg/kg for total BAs in foods for human consumption [12,22]. However, it is very difficult to determine the toxic level of amines since they depend on the existence of other amines and distinctive traits. In the present study, the total BAs levels in farmstead fresh cheeses (*n* = 15) ranged from 11.21 to 62.09 mg/kg (Table 3). The amines cadaverine, tyramine, and spermidine were below detectable levels in all the samples. Only one sample from Halloumi and Cottage cheeses had tryptamine. The string cheese samples merely displayed 2-phenylethylamine, putrescine and histamine (Figure 2a). The 2-phenylethylamine was found predominant in all the samples (*n* = 4) with values between 24.30 and 48.04 mg/kg, exceeding acceptable limit in two samples. The putrescine (3.48 mg/kg) and histamine (13.48 mg/kg) were detected in one sample each, respectively. In all the fresh samples (except string cheese), spermine was dominant ranging from 11.21 to 20.47 mg/kg. The values were slightly higher in Halloumi cheese samples produced from goat’s milk compared to that of cow’s milk. Since spermine is a natural polyamine, their prevalence in cheeses is associated mainly with the initial composition of free amino acids in milk from the ruminants [11]. The total BA content was found highest in a string cheese (62.09 mg/kg), while lowest (11.21 mg/kg) in a Halloumi cheese. The sum of BA levels in all the farmstead fresh cheeses were <100 mg/kg, revealing they are safe for consumption. The mandatory use of pasteurized milk for the cheese production in dairy farms and non-ripening conditions can be the reason for their lower BA content, which can be detoxified by the enzyme monoamino oxidase in the human gut [23]. 

The total BA levels in the farmstead ripened cheeses (hard—Cheddar; semi hard—Gouda) used in this study were found between 257.71 and 384.33 mg/kg (Table 3). The individual and total BA contents in cheeses gradually elevate upon the ripening period. Ripening and proteolysis processes are the most relevant traits that influence the production of BAs in cheeses. The proteolytic activity upsurges upon the ripening ages, contributing to increased free amino acid availability that favors the decarboxylic reactions to eventually accumulate BA [3,24]. Out of the eight tested amines in a Cheddar sample, five (tryptamine, 2-phenylethylamine, tyramine, spermidine, and spermine) were detectable, of which tyramine (82.63 mg/kg) was dominant (Figure 2b). Whereas in Gouda, except putrescine all other tested amines were detectable. The 2-phenylethylamine, cadaverine, histamine, and tyramine were detected in all the samples. The tolerable limit in histamine exceeded in one sample (111.18 mg/kg). Tryptamine and spermidine were seen in two and one out of four samples, respectively. The spermine was found in all the samples ranging from 98.76 to 141.33 mg/kg.

Remarkably, the sum of BA levels in cheddar cheese (257.71 mg/kg) ripened for a period of 5 years was comparatively lower than that of Gouda samples (292.79 to 384.33 mg/kg) having shorter ripening periods (approx. 1 year), which could be related to the activity by non-starter LAB or contaminated microflora in the production farms [6,25]. BA levels exceeding 200 mg/kg in short-time ripening cheeses were previously reported by Bunkova et al. [26]. The total BA levels in all the farmstead ripened cheeses tested in this study was within the maximum permissible limits (<900 mg/kg). Although they seem to be safe for consumption, the presence of potential toxic amines (histamine and tyramine) along with their potentiators (cadaverine) assures the indication of poor hygiene in the production/processing units. Implementation of quality assurance in production process and regular monitoring of proper hygienic practices in the domestic farms can considerably reduce the BA contents in cheeses [27,28]. 

#### 2.1.2. BAs in Imported Fresh and Ripened Cheeses

The overall amine levels in the imported fresh cheeses (*n* = 8) ranged from 7.24 to 125.07 mg/kg (Table 4). Apart from spermine, all other amines were below detectable range in Burrata (*n* = 1) and Mascarpone (*n* = 3) cheeses. In cream cheese (*n* = 2), one sample showed spermine alone, while other one holds five (2-phenylethylamine, putrescine, cadaverine, tyramine, and spermine) out of the eight tested amines. The manifestation of cadaverine at a level of 41.07 mg/kg signifies poor hygienic conditions in production or storage [28]. In ricotta (*n* = 2), 2-phenylethylamine/cadaverine and spermine were determined in one sample each, respectively. The spermine levels in the fresh cheeses varied from 7.24 to 24.31 mg/kg. The total BA levels were attained <25 mg/kg in all the samples, except in a cream cheese (125.07 mg/kg). The lower levels in fresh cheeses are likely due to unripened conditions or the existing microflora does not have adequate time to produce BAs at greater intensities [26]. Overall, the individual and total BA levels in the imported fresh samples were under the permissible limits, suggesting they are safe for consumption. 

The individual and total BA in the imported ripened [hard (h), semi-hard (sh), and soft (s) type] cheeses (*n* = 32) are presented in Table 4. Out of the 32 ripened samples, tryptamine was detectable only in seven (Asiago (h), Mimolette (h), Cheddar (sh), Gruyere (sh), and Feta (s)), with levels varying from 8.07 to 33.27 mg/kg. The 2-phenylethylamine was detected in 44% of ripened samples with a maximum of 22.25 mg/kg in Camembert (s) cheese, which is in the acceptable limit. In absence of tyrosine availability, tyrosine decarboxylase produced by LAB utilizes phenylalanine as a substrate to produce 2-phenylethylamine [29].

**Figure 3:**
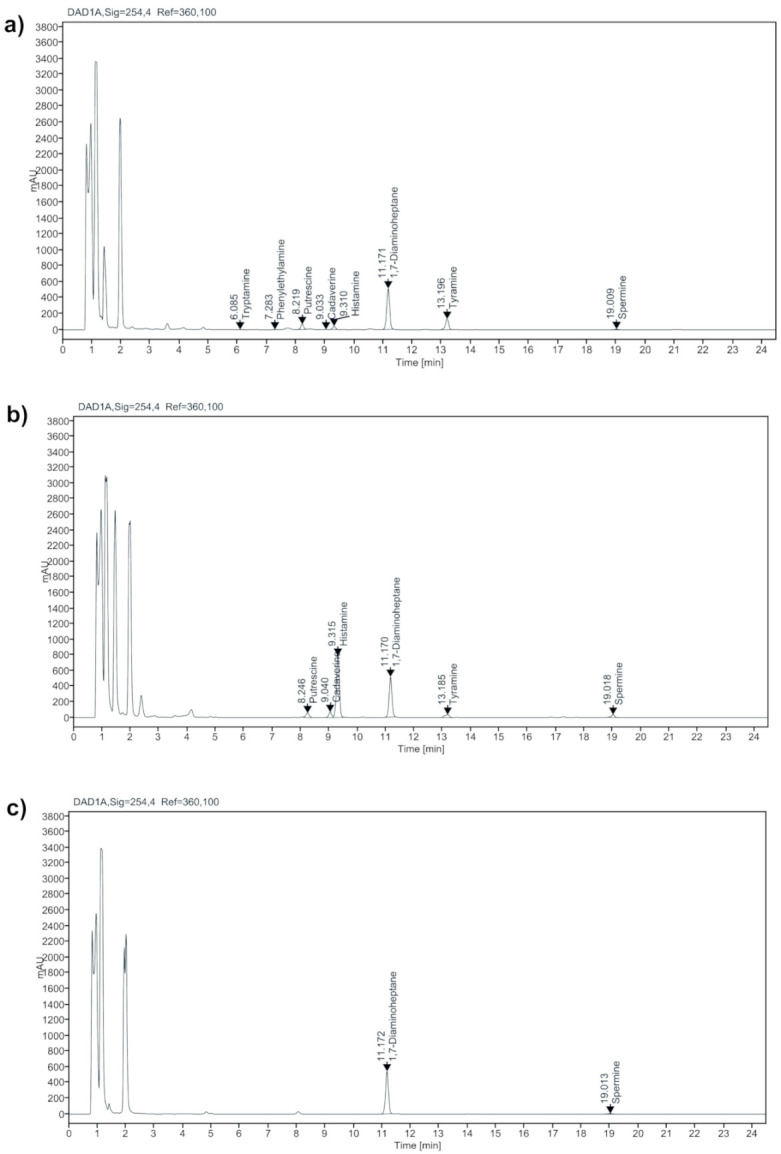
Representative chromatographic separation of biogenic amines in the imported ripened cheeses of Feta (**a**), Pecorino Romano (**b**), and Chevre (**c**) from the retail markets in the Republic of Korea.

Putrescine and cadaverine were present in 63% ripened samples, with values ranging from 6.92 to 211.89 mg/kg and 4.13 to 127.83 mg/kg, respectively. Although these aliphatic diamines gain less importance in pharmacological activity, their assessment is crucial since they contribute to synergistic effects in food and enhancing the toxicity of aromatic amines. Their presence also signifies extended protein degradation and spoilage [7]. In this study, the putrescine level in all the ripened cheeses (hard, semi-hard, and soft-ripened) were below the permissible level (180 mg/kg) [30], except in a Feta sample (211.89 mg/kg, Figure 3a). Marino et al. [27] and Innocente and Agostin [31] associated putrescine with the members of Enterobacteriaceae that serves as indicators of poor quality or hygiene in cheese production units. In case of cadaverine, it was detectable in all the hard and semi-hard cheeses, while only in 2 out of 8 cheeses in soft-ripened type. All the samples hold an acceptable level of <540 mg/kg [12]. Cadaverine is mostly notable at higher amounts in overripened cheeses, and in cheeses produced using raw milk, thus imparting an undesirable flavor [28]. Few food borne-pathogens of lactobacilli strains and *S. thermophiles* are reported to produce cadaverine in food products [32,33]. 

Histamine is the most potent BA with a maximum tolerable level of 100 mg/kg. It is most commonly associated with food-borne intoxication in humans [34]. This amine was detected in 53% of the imported ripened cheese samples ranging from 4.30 to 1468.46 mg/kg. Among the detected samples, Pecorino Romano (1468.46 mg/kg), Grana Padano (1068.51 mg/kg), Appenzeller (225.69 mg/kg), and Cheddar (176.87 mg/kg) exceed the acceptable limit indicating the significant relevance of histamine in the safety assessment of cheese. Pecorino cheeses are most frequently reported for their high levels of histamine accumulation [35]. The histamine levels in the ripened sample were >1000 mg/kg in hard cheese types and >150 mg/kg in semi-hard cheese types. Such histamine levels in hard cheeses were reported previously from several developed countries [8,35]. Only in soft-ripened cheeses, the levels were below tolerable limits from 4.30 to 83.96 mg/kg. In general, histamine intake of 5–10 mg can affect sensitive people and 10 mg in food is a tolerable limit for the body. However, 40–100 mg is considered inducing medium toxicity, and >100 mg as highly toxic [12]. Histamine contamination in dairy foods occurs via certain lactic acid bacteria (LAB) natives related to milk or by the starters/contaminants involved in cheese production during handling or storage [1,4,36]. Upsurging of these microbial loads and consequent histamine accumulation hinges on various environmental (pH, substrate availability, temperature etc.) and technological (pasteurization, proteolysis, ripening, etc.) factors [6,8,11,14,37]. These reports confirm the prevalence of histamine-producing bacteria in the production/processing environments are solely responsible for histamine accumulation in cheeses.

Tyramine, the most common biogenic amine in fermented milk products capable of causing food poisoning symptoms, was detected in 88% samples, widely ranging from 11.08 to 310.11 mg/kg. The term “cheese-reaction” was described as intoxication after the intake of cheese holding higher levels of tyramine. The tyramine levels in all the hard cheeses were <200 mg/kg, as recommended in certain countries. Three samples including Feta (310.11 mg/kg), Edam (209.20 mg/kg), and Cheddar (208.39 mg/kg) cheeses surpassed the limit. The higher levels can be related with the availability of free tyrosine, that is further decarboxylated by tyramine-producers, especially lactic acid bacteria [1,4,9,16]. Ingestion of food containing 50 mg histamine and 600 mg tyramine by a healthy individual does not cause any undesirable health signs, while such amounts in an individual with weak metabolism or administering drugs of monoamine oxidase inhibitors can develop severe poisoning or even death. Concerning the impact of biogenic amines on human health, both histamine and tyramine are counted as the major toxic BAs, while further presence of putrescine and cadaverine potentiate the toxicity of the previous ones [2,7,12]. 

Spermidine was found in nearly 50% samples with a maximum of 26.65 mg/kg, which seems to be much lower compared with all other tested amines. The amine was undetectable in most of the hard and semi-hard cheeses, while detectable in all the soft ripened cheeses. As stated above, spermine was determined in all samples ranging between 11.28 and 153.23 mg/kg. Remarkably, among the tested biogenic amines presence of polyamines alone was observed in Blue (spermidine and spermine) and Chevre (spermine) cheeses. These amines do not originate through the microbial decarboxylation of amino acids, but transfer from milk to the cheese [11].

The cumulative BA levels in the imported ripened cheeses vary widely from 11.28 to 1889.75 mg/kg. Several studies revealed remarkable wide ranges from undetectable to significantly higher levels within a single cheese type. The reasons for this high variability are diverse, ranging from milk composition, pasteurization, degree of proteolytic activity, existence of decarboxylase microflora and expression of the enzyme, length of ripening time, cheese-making process, and their environment [6,13,14]. Concerning the lower tolerable limit (200 mg/kg) of toxic BA levels, about 50% of the imported ripened cheeses are able to cause severe health issues even in healthy individuals. This limit could be altered by the presence or absence of other BAs. The extreme levels of total BAs and especially higher tyramine, histamine, putrescine, and cadaverine levels in the cheese samples certainly set a threat to the consumer. This is caused not only by the high levels of total biogenic amines but also by putrescine and cadaverine that act as potentiators for histamine and tyramine toxicity. Except in Parmigiano Reggiano (126 mg/kg), the total BA levels in other hard cheese samples exceed the acceptable limit. The total BAs even exceeds >1000 mg/kg in case of Pecorino Romano (1889.75 mg/kg, Figure 3b) and Grana Padano (1237.80 mg/kg) cheeses. Moreover, their histamine content alone accounts to 86 and 77% of the total BA levels, respectively. Consumption of such cheeses holding very high cumulative levels of BA can certainly lead to detrimental effects (e.g., headache, nausea, hypo- or hypertension) [2,5,12]. The total BA in semi-hard cheeses showed a wide variation from 35.81 to 588.14 mg/kg. Nearly 57% samples in semi- hard type cheeses exceed the acceptable limit (200 mg/kg), with maximum levels (>500 mg/kg) in Cheddar (588.14 mg/kg) and Appenzeller (552.45 mg/kg) cheeses prevalent with larger histamine, tyramine and spermine amounts. Spermine along with tyramine increases the total BA levels in Gouda, Emmental, Smoked, Comte, and Edam cheeses. In Gruyere cheese, the individual spermine level alone accounts for 50% (>100 mg/kg) of the total levels (>200 mg/kg). Apart from Feta and Fauquet Maroilles cheeses, the total BA levels in the other soft-ripened cheeses (Camembert, Brie, Chevre, Caprice des Dieux, Bleu d’Auvergne and Blue) were within the acceptable limit (<200 mg/kg). The feta (*n* = 2) cheese samples comprised of all the tested amines with total BA levels of >500 mg/kg influenced by larger tyramine and putrescine (>100 mg/kg), and lower tryptamine and 2-phenylethylamine levels. These results are in concomitant to the earlier studies by Valsamaki et al. [37]. Spermine along with tyramine increases the total levels in Fauquet Maroilles (274.86 mg/kg), while spermine alone in Caprice des dieux and Bleu d’Auvergne cheeses. In Blue cheese, the polyamine alone contributed to the total BA levels (45.30 mg/kg). The lowest total BA level was found in Chevre cheese (11.28 mg/kg), in which spermine alone was detectable (Figure 3c). Surprisingly, the higher and lower total BA levels in this study were found in cheeses produced using non-cow milk (sheep and goat), respectively. The reasons for lower amounts of an individual and total BA in few ripened cheese samples could be the absence of the species/strains that can produce the amines or the ability of certain starter cultures to produce amine oxidase enzymes for the degradation of biogenic amines [6,11]. 

### 2.2. Multivariate Statistical Analysis 

**Figure 4:**
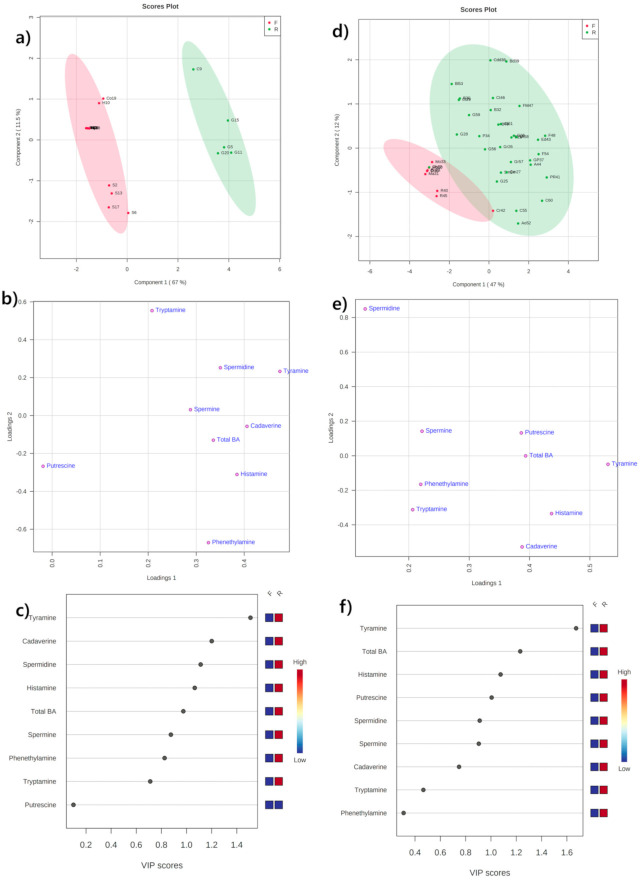
Partial least-squares discriminant analysis (PLS-DA) score (**a**,**d**), loading (**b**,**e**) and VIP plots (**c**,**f**) derived from the biogenic amine profiles demonstrating the separation between the fresh and ripened cheese samples from the farmstead (left) and retail markets (right) in the Republic of Korea.

Multivariate statistical analysis for the BA content in the domestic and imported cheeses was studied using a supervised method, PLS-DA and their results are presented in Figure 4a–c. The score plots (Figure 4a) for the domestic fresh (negative in PC1) and ripened (positive in PC1) cheeses elucidate the position of these groups on opposite side of the axes, signifying higher variations between the groups in their BA content and composition. The combined PLS 1 and PLS2 components explained a total variance of 78.5% (67% and 11.5%, respectively). Among domestic fresh cheeses, the samples of string cheese lie separately and in distant from the Halloumi and cottage samples indicating significant variation in their BA composition. A tight clustering between the Halloumi cheese samples (except one sample) indicates no variation within their composition. A similar trend was also observed in the domestic ripened cheeses, between the samples of gouda and cheddar. In the loading plots (Figure 4b), all amines (except putrescine) lie in the right side (positive in PLS1), indicating their richness in ripened samples than fresh ones. The amines tryptamine, tyramine, spermidine, and spermine positive in PLS1 and PLS2 axis were abundant in Cheddar cheese. While cadaverine, histamine, and 2-phenylethylamine, found positive in PLS1 and negative in PLS2 axes, were dominant in Gouda samples. The putrescine found at distant and negative in both axes relates their absence in all the domestic samples. Subsequent VIP plots (Figure 4c) from the PLS-DA explains tyramine, cadaverine, spermidine, histamine, and total BA content are the major variables (VIP values ≥ 1) involved in the separation of the domestic fresh and ripened samples. The R2 and Q2 intercept values were 0.979 and 0.934, respectively with a significant value of *p* < 0.05 after 20 permutations. 

The PLS-DA model on the imported fresh and ripened cheeses from the retail markets explained a total variance of 59%, with 47% and 12% in PLS1 and PLS2 (Figure 4d–f) components, respectively. The score plot (Figure 4d) displays a slight overlapping among the fresh and ripened cheeses, although the fresh samples were completely on negative side of both PLS1 and PLS2. Among the fresh samples, cream cheese (Cr42) sample accumulating higher total BA (125.07 mg/kg) lies in a distant and at boundary of the Hoteling’s T2 ellipse. The ripened cheese samples were well scattered around the ellipse, indicating variation within their composition. Only the chevre sample was found to lie outside the ellipse of ripened samples and clustered within the fresh cheeses, which might be due to their lower levels of total BA (11.28 mg/kg). The loading plot (Figure 4e) displays the variable spermidine is distant from other variables, because of their absence in all the fresh samples and in 50% of the ripened cheese samples. The location of the other variables indicates their presence with respect to the cheese samples’ positions in the score plot. The VIP plot (Figure 4f) indicates the major variables tyramine, total BA, histamine, putrescine, spermidine, and spermine are involved in the separation. The R2 and Q2 intercept values were 0.673 and 0.467, respectively with a significant value of *p* < 0.05 after 20 permutations. 

**Figure 5:**
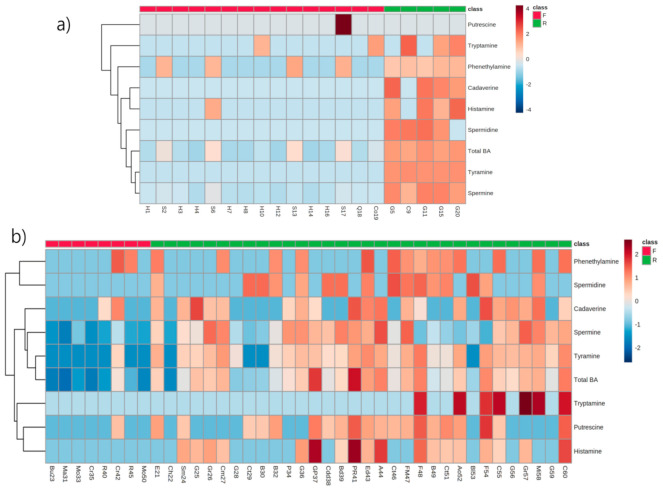
Heat map profiles of the fresh and ripened cheese samples from the farmstead (**a**) and retail markets (**b**) in the Republic of Korea.

Further heatmaps on the farmstead and imported cheeses display clear differentiation between the fresh and ripened groups and also the variation in the proportion of each amine in the cheese samples used in this study (Figure 5). The heat maps not only visualize a clear discrimination in the individual and total amine content among the different cheese types, but also within the same type.

## 3. Materials and Methods

### 3.1. Sample Details

Cheese samples (*n* = 60) were obtained from various domestic farms and retail markets in the ROK during the year, 2019 and assorted into four groups as domestic fresh, domestic ripened, imported fresh, and imported ripened cheeses. The samples were transported to the work place using portable coolers (4 ± 1 °C) and immediately homogenized (100 g) and stored at −20 °C. The speed and duration of the homogenization was varied depending upon the cheese type (hard, semi-hard, and soft). All the samples were analyzed within a week after the purchase of the products or before end of shelf-life period. 

### 3.2. Reagents and Materials

The standard stock solution (1000 mg/L) for each of the eight biogenic amines was prepared in ultrapure water using tryptamine hydrochloride, phenylethylamine hydrochloride, putrescine dihydrochloride, cadaverine dihydrochloride, histamine dihydrochloride, tyramine hydrochloride, spermidine trihydrochloride, and spermine tetrahydrochloride. The working solution (100 mg/L) containing the mixture of eight biogenic amines was prepared by diluting the stock solution with 0.4 M perchloric acid. 1,7-Diaminoheptane was used as an internal standard. All chemicals used in study were of HPLC grade and purchased from Sigma Aldrich (Seoul, Korea).

### 3.3. BA Extraction and Derivatisation of Sample Extracts and Mixed Standards

The eight biogenic amines (TRP, PHE, PUT, CAD, HIS, TYR, SPD, and SPM) were detected and quantified using high-performance liquid chromatography equipped with a diode array detector (WR G7115 A, Agilent Technologies, Santa Clara, CA, USA) at 254 nm, as described in Eerola et al. [38]. The extraction of BA’s was carried by homogenizing each cheese sample (5 g) with 20 mL of 0.4 M perchloric acid followed by centrifugation (3000 rpm, 10 min, 4 °C). A second extraction was performed again with 20 mL 0.4 M perchloric acid and both extracts were pooled together and the total volume was made up to 50 mL with 0.4 M perchloric acid. The extract was further filtered through Whatman paper No.1 (0.2 μm).

Before derivatization, the extract/standard solutions (1 mL) were initially alkalinized by adding 200 μL 2M NaOH and 300 μL saturated sodium bicarbonate. To this, 2 mL of dansyl chloride (1% *w*/*v* in acetone) was added and allowed to react in a dark room at 40 °C, 45 min with intermittent stirring. For removal of the residual dansyl chloride, 100 μL of 25% ammonium hydroxide was added to the mixture and kept in room temperature for 30 min. The sample volume was made up to 5 mL with acetonitrile and centrifuged at 3000 rpm for 10 min at 4 °C. The final supernatant was filtered using 0.2 μm membrane filter (Sartorius, Goettingen, Germany) and stored at −25 °C until HPLC analysis. 

### 3.4. HPLC-DAD Analysis

HPLC analysis was performed using a liquid chromatography (Agilent 1260 infinity II Series HPLC, Santa Clara, CA, USA) equipped with a 1260 DAD UV/Vis detector (WR G7115A) according to Eerola et al. [38] and Ben-Gigigrey et al. [39]. The chromatographic separation was done using Nova-Pak C18 column (particle size 4 μm, 3.9 × 150 mm; Waters, Milford, MA, USA) at 40 °C, with an injection volume of 20 μL. The mobile phase comprised of 0.1 M ammonium acetate (Solvent A) and 100% acetonitrile (Solvent B) processed in a gradient mode at the flow rate of 1 mL/min [38]. For the gradient, the initial was set at 50% acetonitrile and raised to 90% after 19 min (Table 5). Revert to initial settings was achieved in one minute and equilibrated for 10 min until the next run. The compounds were quantified using internal calibration curves plotted for each BA and expressed as mg/kg (wet weight). 

### 3.5. Statistical Analysis

All the cheese samples in this study were analyzed in duplicates and the BA content stated herein thereby represents the means ± standard deviation of two parallel analyses. Multivariate statistical analysis such as partial least-squares discriminant analysis (PLS-DA) and hierarchical cluster analyzing (HCA) heatmaps were performed in the webserver MetaboAnalyst 4.0 [40].

## 4. Conclusions

In this study, the content of eight BAs and their sum were analyzed in the domestic and imported fresh and ripened cheeses available in the retail markets of the Republic of Korea. A wide variation in both the individual and total amine contents was observed among all the cheese samples, which could be related with several factors such as the hygienic quality of raw materials, methods of handling and cheese-making procedures, degree of ripening that could support the growth and activity of decarboxylase microorganisms involved in synthesis of BAs. Compared with fresh samples, the ripened cheeses accumulated higher BA content because of the ripening periods. Among the ripened cheeses, the hard ripened ones showed the greatest contents, followed by semi-hard and soft ripened cheeses. The total BA levels in Grana Padano and Pecorino Romano surpassed >1000 mg/kg. While the potent histamine and tryramine levels exceeded their tolerable limits in few cheese samples. Consumption of such cheeses represents a possible risk on consumer health according to the legislation or literature reports stated earlier. Moreover, cheeses are commonly served with alcoholic drinks (beer or wine) which decrease the detoxifying activity of the intestinal enzymes [26], hence, even the cheeses having acceptable limits can produce adverse health effects. Since there is no safe regulation on the BA levels for cheeses in the Republic of Korea, the contents are not examined or assessed by any regulatory body. Thus, statutory monitoring of BAs in cheese industries throughout the production process, adoption of strict hygiene measures, and regulation of BA content is mandatory to confirm the high quality and safety of the consumers on all cheese products in future.

## Figures and Tables

**Table 1:** Results of the validation parameters for the biogenic amines in HPLC.

Biogenic Amines	Linearity (r^2^)	LOD (mg/kg)	LOQ (mg/kg)
TRP	0.992	0.38	1.25
PHE	0.993	0.34	1.12
PUT	0.997	0.16	0.53
CAD	0.996	0.16	0.55
HIS	0.996	0.20	0.65
TYR	0.996	0.29	0.95
SPD	0.997	0.22	0.73
SPM	0.995	0.22	0.74

LOD—limit of detection; LOQ—limit of quantification; TRP—tryptamine, PHE—2-phenylethylamine, PUT—putrescine, CAD—cadaverine, HIS—histamine, TYR—tyramine, SPD—spermidine, and SPM—spermine.

**Table 2:** Distribution of biogenic amines (mg/kg) in cheeses from the farmstead and retails markets in the Republic of Korea.

Cheese & Type	Number	Samples	Biogenic Amines Detected (%) ^a^							
			TRP	PHE	PUT	CAD	HIS	TYR	SPD	SPM
**Domestic**										
Fresh	4	15	2 (13.3%)	4 (26.7%)	1 (6.7%)	ND	1 (6.67%)	ND	ND	15 (100%)
Ripened										
*Hard/Semi-hard*	2	5	3 (60%)	5 (100%)	ND	4 (80%)	4 (80%)	5 (100%)	5 (100%)	5(100%)
**Imported**										
Fresh	4	8	ND	2 (25%)	1(12.5%)	2(25%)	ND	1(12.5%)	ND	8(100%)
Ripened										
*Hard*	5	5	2 (40%)	2 (40%)	3(60%)	5(100%)	3(60%)	5(100%)	1(20%)	5(100%)
*Semi-hard*	8	14	3 (21.4%)	5 (35.7%)	6(42.9%)	12(85.7%)	14(100%)	13(92.9%)	3(21.4%)	14(100%)
*Soft-ripened*	8	13	2 (15.4%)	6 (46.2%)	11(84.6%)	3(23.1%)	5(38.5%)	13(100%)	3(23.1%)	13(100%)

Abbreviations for TRP—tryptamine, PHE—2-phenylethylamine, PUT—putrescine, CAD—cadaverine, HIS—histamine, TY—tyramine, SPD—spermidine and SPM—spermine; ND—not determined; ^a^—number of samples in which biogenic amine detected (percentage of samples in which biogenic amines detected).

**Table 3:** Total and individual biogenic amine contents in cheese samples from the farmsteads in the Republic of Korea.

Cheese	Milk	Biogenic Amines (mg/kg) ^a^								
		TRP	PHE	PUT	CAD	HIS	TYR	SPD	SPM	Total
**Fresh** (*n* = 15)										
Halloumi (*n* = 9)	Goat (*n* = 2)	ND	ND	ND	ND	ND	ND	ND	2/15.19–15.71	15.19–15.71
	Cow (*n* = 7)	1/ND–5.92	ND	ND	ND	ND	ND	ND	7/11.21–13.16	11.21–18.96
String (*n* = 4)	Cow	ND	4/24.30–48.04	1/ND–3.48	ND	1/ND–13.48 ± 0.02	ND	ND	4/14.05–20.47	43.01–62.09
Quark (*n* = 1)		ND	ND	ND	ND	ND	ND	ND	15.10 ± 1.16	15.10
Cottage (*n* = 1)		12.04 ± 2.74	ND	ND	ND	ND	ND	ND	14.80 ± 0.16	26.84
**Ripened** (*n* = 5)										
*Hard* Cheddar (*n* = 1)	Cow	65.19 ± 10.26	16.87 ± 0.04	ND	ND	ND	82.63 ± 16.22	23.17	69.86 ± 13.85	257.71
*Semi-hard* Gouda (*n* = 4)		2/ND–26.20	4/11.89–22.57	ND	4/17.68–92.47	4/9.66–111.18	4/59.34–70.77	3/ND-9.85	4/98.76–141.33	292.79–384.33

Values are mean of two independent determinations ± standard deviation; ^a^—number of samples in which biogenic amines detected/range of detected biogenic amine levels; ND—biogenic amines not determined; *n*—number of samples. Abbreviations for TRP—tryptamine, PHE—2-phenylethylamine, PUT—putrescine, CAD—cadaverine, HIS—histamine, TYR—tyramine, SPD—spermidine, and SPM—spermine.

**Table 4:** Total and individual biogenic amine contents in cheese samples from the retail markets in the Republic of Korea.

Cheese	Milk	Biogenic Amines (mg/kg) ^a^								
		TRP	PHE	PUT	CAD	HIS	TYR	SPD	SPM	Total
**Fresh (*n* = 8)**										
Burata (*n* = 1)	cow	ND	ND	ND	ND	ND	ND	ND	9.27 ± 3.42	9.27
Mascarpone (*n* = 3)		ND	ND	ND	ND	ND	ND	ND	3/7.24–15.87	7.24–15.87
Cream (*n* = 2)		ND	1/ND–24.47	1/ND–9.57 ± 0.75	1/ND–41.07 ± 1.07	ND	1/ND–25.66 ± 1.46	ND	2/8.85–24.31	8.85–125.07
Ricotta (*n* = 2)		ND	1/ND–12.12	ND	1/ND–4.16	ND	ND	ND	2/11.16–11.55	15.32–23.67
**Ripened** (*n* = 32)										
*Hard*										
Pecorino romano (*n* = 1)	Sheep	ND	ND	99.43 ±2.05	108.20	1468.46 ± 24.22	125.02 ± 9.16	ND	88.65 ± 13.16	1889.75
Parmigiano-Reggiano (*n* = 1)	Cow	ND	ND	ND	5.07 ± 2.24	ND	31.81 ± 4.55	ND	90.11 ± 2.20	126.99
Grana Padano (*n* = 1)		ND	ND	49.84 ±5.55	4.13	,068.51 ± 12.93	48.36 ± 11.73	ND	66.95 ± 13.11	1237.80
Asiago (*n* = 1)		14.08 ± 3.99	15.02 ± 1.44	23.06	18.27 ± 6.03	7.92	110.93 ± 4.34	ND	15.53 ± 2.29	204.81
Mimolette (*n* = 1)		17.88 ± 0.33	16.75	ND	64.19	ND	96.32 ± 1.19	8.71	104.01 ± 31.53	307.86
*Semi Hard*										
Gouda(*n* = 5)	Cow	ND	1/ND–9.63 ± 0.20	ND	3/ND–152.92	2/ND–24.15	4/12.70–57.27	ND–5.25	4/23.11–91.91	35.81–230.69
Cheddar (*n* = 2)		2/14.67–11.38	2/15.94–17.79	2/7.86–91.44	2/5.95–18.88	2/4.68–176.87	2/63.03–208.39	ND	2/56.34–78.17	183.25–588.14
Gruyere (*n* = 2)		1/ND–33.27	ND	ND	2/9.35–17.04	2/11.41–22.25	2/52.47–75.89	ND	2/125.53–133.24	209.60–270.85
Emmental (*n* = 1)		ND	12.63	22.64 ± 11.50	13.26	ND	81.98 ± 34.79	4.27	50.48 ± 4.68	185.26
Smoked (*n* = 1)		ND	ND	8.22	13.06 ± 0.19	ND	30.99 ± 2.04	ND	42.74 ± 1.53	95.01
Comte (*n* = 1)		ND	10.03 ± 0.34	ND	10.27 ± 0.65	8.69 ± 0.85	149.13 ± 34.58	ND	101.29 ± 0.54	279.41
Edam (*n* = 1)		ND	26.56 ± 3.57	20.61	41.83 ± 4.31	12.79	209.20 ± 3.37	10.80	73.05 ± 2.21	394.84
Appenzeller (*n* = 1)		ND	ND	13.77 ± 3.47	58.08	225.69 ± 72.76	101.67 ± 5.63	ND	153.23 ± 28.78	552.45
*Soft Ripened*										
Camembert (*n* = 3)	Cow	ND	2/ND–22.25	3/6.92–37.40	ND	1/ND–7.23	2/ND–11.53	3/10.23–26.65	3/15.20–33.12	38.03–105.00
Brie(*n* =3)		ND	2/ND–9.90	3/7.43–23.82	ND	1/ND–8.67 ± 2.23	2/ND–12.81	3/7.44–15.87	3/17.12–34.20	40.42–88.39
Feta (*n* = 2)	Goat	2/8.07–9.82	1/ND–5.22	2/100.05–211.89	2/3.00–127.83	2/14.27–83.96	2/129.10–310.11	2/5.88–17.35	2/12.80–15.22	509.84–544.72
Chevre (*n* = 1)		ND	ND	ND	ND	ND	ND	ND	11.28 ± 2.58	11.28
Caprice des dieux (*n* = 1)	Cow	ND	ND	8.01 ± 3.54	ND	ND	19.40 ± 13.59	14.39	59.97 ± 18.56	101.77
Bleu d’Auvergne (*n* = 1)		ND	ND	13.57 ± 5.26	ND	4.30	22.93 ± 21.92	14.70 ± 4.29	105.99 ± 82.75	161.50
Fauquet Maroilles (*n* = 1)		ND	8.90	15.35 ± 11.68	14.18 ± 1.47	ND	90.66 ± 23.14	13.67	132.11 ± 5.29	274.86
Blue (*n* = 1)		ND	ND	ND	ND	ND	ND	25.10 ± 5.40	20.20 ± 0.16	45.30

Values are mean of two independent determinations ± standard deviation; ^a^—number of samples in which biogenic amines detected/range of detected biogenic amine levels; ND—biogenic amines not determined; *n*—number of samples. Abbreviations for TRP—tryptamine, PHE—2-phenylethylamine, PUT—putrescine, CAD—cadaverine, HIS—histamine, TYR—tyramine, SPD—spermidine, and SPM—spermine.

**Table 5:** Gradient elution program (Flow rate 1 mL/min).

Time (min)	A-0.1 M Ammonium Acetate (%)	B-100% Acetonitrile (%)
0	50	50
19	10	90
20	50	50
25	50	50

## Data Availability

All data are provided in the manuscript.
